# Effect of gymnastics on balance ability in children aged three to six years

**DOI:** 10.3389/fpsyg.2025.1549741

**Published:** 2025-03-04

**Authors:** Qiaoyan Yu, Xiaofei Pan, Zijing Liu, Chenliang Deng

**Affiliations:** ^1^School of Physical Education, Chengdu Sport University, Chengdu, China; ^2^Sports Teaching and Research Group, Chengdu Jinxi Middle School, Chengdu, China; ^3^Sports Department, University of Electronic Science and Technology of China, Chengdu, China

**Keywords:** 3–6 year old children, gymnastics movements, balance system training, core functional training, balance ability

## Abstract

**Objective:**

This study aims to explore the intervention effect of gymnastics movements on children’s balance ability.

**Methods:**

The study selected 24 healthy children aged 3–6 years and randomly divided them into a control group and an experimental group for a 12 week experimental intervention. The experimental group conducted scientific gymnastics exercises, including proprioceptive training, single foot static balance training, and dynamic balance training; the control group maintained a normal sports lifestyle. Static and dynamic balance were assessed using methods from the National Physical Fitness Testing Standards Manual, and data were compared before and after the experiment.

**Results:**

The research results showed that the static and dynamic balance of the experimental group children were significantly improved, and the improvement effect was significantly better than that before the experiment and the control group (*p*<0.01). There was no significant difference in the control group before and after intervention (*p*>0.05), while there was a very significant difference in the experimental group before and after intervention (*p*<0.01). In addition, there is a difference in balance ability between boys and girls (*p*<0.05), with girls having better static balance ability and boys having stronger dynamic balance ability.

**Discussions and conclusions:**

The research conclusion is that long-term scientific gymnastics exercises can significantly improve children’s balance ability, and the effect is better than irregular physical exercise. It is suggested that government departments formulate policies to promote the promotion of gymnastics among young children. Schools should use gymnastics movements as the main method to improve children’s balance ability, and combine them with games to enrich teaching methods and attract children to participate. Parents should encourage their children to practice gymnastics or receive professional training while ensuring safety, in order to promote the healthy growth of young children. This study provides a scientific basis for improving children’s balance ability and enriches the value theory of gymnastics movements.

## 1 Introduction

In the physiological structure of the human body, the maintenance of balance relies on the synergistic effect of the vestibular system, visual system, and proprioceptive system (Wei et al., 2022). These three systems work together to enable individuals to accurately perceive their own movement status, the position of their head in space, and the external environment, thereby ensuring stable and coordinated posture and movement (Hu et al., 2022). For young children, the development of balance ability is particularly important, as it not only relates to the mastery of basic motor skills, but also directly affects their future physical coordination, athletic performance, and even daily living abilities.

The 3–6 year old is a critical period for the rapid physical and mental development of young children. During this stage, their nervous system gradually matures, their motor skills rapidly improve, and their balance ability is also in a stage of rapid development. However, changes in modern lifestyles, such as the popularization of electronic products and the reduction of outdoor activities, may to some extent affect the normal development of children’s balance ability. Therefore, it is particularly important to find effective intervention methods to promote the healthy development of children’s balance ability.

Gymnastics movements, with their rich content and diverse forms, have become an effective means of improving the balance ability of teenagers (Wu et al., 2004). The rotation, jumping, rolling, posture changes, posture adjustments, and spatial position perception in gymnastics require practitioners to constantly adjust the position of their head and body in order to exercise and improve their balance ability (Wu and Fu, 2024). For young children, gymnastics exercises not only have fun and can stimulate their interest in sports, but also subtly improve their balance ability and promote their physical and mental health development through play (Ma, 2001; Li, 2021).

The aim of this study is to explore the intervention effect of gymnastics movements on the balance ability of 3–6 year old children. By designing a scientifically reasonable gymnastics training program, the changes in balance ability of children before and after training will be observed and analyzed. The study will combine the physiological mechanisms of the vestibular system, visual system, and proprioceptive system to deeply analyze the specific pathways through which gymnastics movements enhance children’s balance ability, providing scientific basis for improving children’s balance ability. At the same time, this study will further enrich the value theory of gymnastics movements, providing new ideas and methods for fields such as early childhood education and physical training.

## 2 Methods

### 2.1 Research design

From September 2024 to November 2024, 24 healthy children aged 3–6 (12 males and 12 females) were selected from the physical training center of Chengdu Shanda Fitness Service Co., Ltd. in China. They were randomly divided into a control group and an experimental group for a 12 week experimental intervention. By asking the parents or guardians of the child and combining their birth certificate or relevant documents to determine the exact age of the child, this process does not involve specific measuring tools or brands. Use the Beya Beryl height ruler (model: BYHJ02) for height measurement. When measuring, ask the child to take off their shoes and stand upright on the measuring instrument, with their head, hips, and heels tightly attached to the measuring ruler. The measuring personnel should ensure that the ruler is perpendicular to the ground before reading the data. We use the Xiaomi intelligent weight scale (model: MMS-041) for weight measurement. Before measurement, ensure that the surface of the weight scale is clean and free of foreign objects. Let the child stand in the center of the scale wearing lightweight clothing and record the data after the reading stabilizes. During all measurements, 1–2 researchers are present, with one responsible for operating the measurement tool and the other responsible for recording and verifying the data to ensure accuracy and consistency of the measurements. In addition, all measuring tools have been properly calibrated and maintained before use to ensure the reliability of the measurement results. Through relevant data analysis, it was ensured that there were no statistically significant differences (*p* > 0.05) in age, height, and BMI between the two groups, providing a reasonable foundation for subsequent experimental interventions, see Table 1. Through relevant data analysis, there was no statistically significant difference in age, height, and weight between the two groups (p > 0.05). The experimental group trains three times a week for one hour each time, scheduled from 19:00–20:00 on Monday, Wednesday, and Friday evenings. The training process is divided into a warm-up part (10 min), a training part (45 min, including rest), and a relaxation part (5 min). Based on previous research results and expert suggestions, the experimental content includes standing with closed eyes and walking on a balance beam (Jafari and Malayeri, 2011), practicing in different positions, lifting with one foot (Toit and Pienaar, 2001), rolling over (Wang et al., 2016), trampoline (Giagazoglou et al., 2013) and other gymnastics movements. Training is divided into three stages, and the specific implementation of each stage is as follows:

Phase 1: Proprioceptive training (Zhang et al., 2022). Training content: Practice lying with eyes open, sitting, standing, standing with both feet closed, and standing on one foot on a balance board, tilting left and right, and forward and backward. Action standard: Ensure that children maintain physical stability during practice, pay attention to correct posture, and avoid excessive tilting or falling. Training intensity: 2–3 groups per session, 3–6 sessions per group, with a 30 s break between groups. According to individual differences among children, adjust the number of exercises and groups appropriately to ensure the safety and effectiveness of training. Standardization and Consistency: All training is conducted by the same coach to ensure standardization and consistency of training movements. The coach will closely monitor the children’s reactions during the training process and adjust the training intensity in a timely manner.

Phase 2: Single foot static balance training (Wu et al., 2014). Training content: Lift one foot and maintain body stability while performing sitting, standing, leaning, elbow bending, and other movements on a balanced soft step. Action standard: Ensure that the lifted foot does not touch the ground as much as possible, and the foot in contact with the ground should maintain stability. The coach will provide necessary assistance and protection to ensure the safety of the children. Training intensity: 2–3 groups per session, 10–20 sessions per group, with a 30 s break between groups. Gradually increase the number and difficulty of exercises based on the child’s progress. Standardization and Consistency: Similar to the first stage, ensure the standardization and consistency of training movements, and the coach will make adjustments based on individual differences of children.

Phase 3: Dynamic balance training (Yu et al., 2020). Training content: Practice in interference environments, on balance beams, gymnastics mats, and trampolines, including single foot standing to receive balls, protective pushing and pulling on both feet, protective stepping in front, back, left, and right directions, balance beam exercises (alternating walking forward with both feet, lateral movement walking, crawling, etc.), gymnastics mat exercises (roller, forward roll, backward roll, etc.), and free jumping exercises on trampolines. Action standard: Ensure that children maintain physical stability during practice, pay attention to the correctness and safety of posture. The coach will provide necessary assistance and protection to prevent children from getting injured. Training intensity: 3–6 groups per session, 6–12 sessions per group, with a 30 s break between groups. Gradually increase the number and difficulty of exercises based on the child’s progress and physical fitness level. Standardization and Consistency: Ensure the standardization and consistency of training movements in the previous two stages. The coach will make timely adjustments based on the individual differences and training progress of the children.

**Table 1:** General information comparison of research subjects ( $\bar{X}$ ±SD).

Group	Control group (*n*=12)	Experimental group (*n*=12)	*t*	*P*
Age/yr	4.23 ± 0.79	4.21 ± 0.83	-0.075	0.878
Height/cm	102.29 ± 6.00	102.71 ± 6.12	0.168	0.945
BMI/(kg/m2)	14.92 ± 1.14	15.09 ± 1.18	0.369	0.912

Throughout the entire training process, the coach will closely monitor the individual differences of children, including physical fitness level, coordination ability, reaction speed, etc. According to the actual situation of children, coaches will adjust the training intensity, difficulty of movements, and number of exercises in a timely manner to ensure the safety and effectiveness of training.

The control group of children did not participate in the regular gymnastics exercises conducted by the experimental group, but maintained their normal physical lifestyle. In order to ensure the rigor of the experiment and the traceability of the control group’s activities, we have set requirements for the daily physical activities of the control group, requiring parents to supervise or accompany them as much as possible, and to communicate and understand the situation at any time. The specific requirements are as follows: In terms of exercise frequency, the control group of children will maintain at least 3 physical activities per week, which matches the training frequency of the experimental group, in order to form an effective comparison in frequency. In terms of sports types, the control group of children will mainly engage in outdoor free play (such as running, chasing games, climbing, etc.), indoor parent–child games (such as passing, skipping rope, dancing, etc.), and natural sports in daily activities (such as going up and down stairs, walking, etc.). These types of sports aim to reflect the various forms of exercise that young children may encounter in their daily lives, while avoiding the introduction of gymnastics exercises unique to the experimental group. In terms of exercise intensity and duration, each physical activity of the control group children will last for about 30–60 min, and the specific intensity and duration will be naturally adjusted according to the children’s interests and physical condition. Through the above settings, we can have a clearer understanding of the specific activities of the control group during the experiment, and thus more accurately evaluate the specific impact of gymnastics exercises taken by the experimental group on the development of children’s balance ability.

### 2.2 Balance ability test

This study selected the balance ability testing methods for students based on the “National Physical Fitness Testing Standards Manual” compiled by the General Administration of Sport of China, including standing on one foot with eyes open, standing on one foot with eyes closed, and walking on a balance beam to evaluate the static and dynamic balance abilities of young children.

The one legged standing test with open eyes is a test to evaluate balance ability. During the test, the subjects need to stand on a flat ground, keep their hands on their hips upright, and then lift either foot to maintain a single legged standing posture as much as possible until they cannot maintain balance or reach the prescribed time limit. Testers will record the time for subjects to maintain balance, and the longer the time, the better their balance ability. All testers have received professional training, familiarized themselves with the testing process, scoring criteria, and safety precautions to ensure the accuracy of the testing and the safety of the subjects.

The closed eye one foot standing test is a variant of the open eye one foot standing test, which requires participants to perform the test with their eyes closed. The testing procedure is similar to the eye opening test, where the tester will issue a start command after the subject closes their eyes and record the time they maintain balance. The scoring criteria are also based on the time required to maintain balance, with longer time indicating better balance ability. The personnel performing the test have the corresponding professional knowledge and experience, and are able to accurately execute the testing procedure and effectively evaluate the results.

The balance beam walking test requires the subject to walk on a balance beam of a specified width (e.g., 10 centimeters), and the tester will record the time required for the subject to complete the walking and whether there is any loss of balance. The scoring criteria are determined based on the length of completion time and whether balance is maintained. The shorter the completion time and the one who has not lost balance, the better the balance ability. The test should be conducted in a quiet and undisturbed environment to ensure that the balance beam is stable and slip resistant. Throughout the entire testing process, testers will strictly monitor the safety status of the subjects and provide assistance or take protective measures as necessary to ensure the safe conduct of the testing.

All participants in the experiment received professional training before the start of the experiment, including testing procedures, scoring criteria, safety precautions, and emergency response measures. Through training, ensure that testing personnel can accurately and safely perform testing tasks, and improve the reliability and accuracy of testing results.

### 2.3 Data processing

Collect baseline data before the start of the experiment (within 0 weeks) and post intervention data at the end of the 12 week experiment. Perform *t*-test on the test data of the experimental group and control group using SPSS 22.0, with a significance level of *α* = 0.05.

## 3 Results

After 12 weeks of experimental intervention, the static and dynamic balance of the experimental group children were significantly improved, and the improvement effect of children’s balance ability was significantly better than before the experiment and the control group.

### 3.1 Changes in balance ability between experimental group and control group before and after intervention

The experimental results showed that the experimental group of children showed significant improvement in both static balance (standing on one foot with open eyes, standing on one foot with closed eyes) and dynamic balance (walking on a balance beam) (*p* < 0.01), while the control group had no significant difference in balance ability before and after intervention (*p* > 0.05). Compared with the control group, the experimental group showed significant differences in balance ability in all three tests (*p* < 0.01), see Table 2.

**Table 2:** Analysis results of balance ability test.

Test project	Group	*n*	Before intervention (s)	After intervention (s)	*t*	*P*
Standing on one foot with open eyes	Experimental group	12	5.21 ± 1.16	13.16 ± 5.89	-5.187	<0.001
	Control group	12	4.71 ± 0.44	4.73 ± 0.43	-0.561	0.586
	*t*		1.392	4.950		
	*P*		0.178	<0.001		
Standing on one foot with closed eyes	Experimental group	12	4.53 ± 1.27	9.88 ± 3.75	-6.129	<0.001
	Control group	12	4.24 ± 0.45	4.28 ± 0.46	-0.842	0.417
	*t*		0.729	5.130		
	*P*		0.474	<0.001		
Balance beam walking	Experimental group	12	12.25 ± 0.91	7.88 ± 3.08	5.699	<0.001
	Control group	12	12.28 ± 1.20	12.29 ± 1.06	-0.297	0.772
	*t*		-0.058	-4.694		
	*P*		0.477	<0.001		

The *t*-value is the statistical measure of the independent sample and paired sample *t*-test, and the *p*-value is the corresponding significance level.

### 3.2 Analysis of gender differences in balance ability

Further analysis reveals that there are gender differences in the balance ability of young children. In terms of static balance, girls have longer periods of standing on one foot with their eyes open and standing on one foot with their eyes closed than boys, with a significant difference (*p* < 0.05), indicating that girls have better static balance ability. However, in terms of dynamic balance, boys have shorter walking time on the balance beam than girls, with a significant difference (*p* < 0.05), indicating that boys have stronger dynamic balance ability, see Table 3.

**Table 3:** Analysis of gender differences in balance ability.

Test project	Gender	*n*	Before intervention (s)	*t*	*P*	After intervention (s)	*t*	*P*
Standing on one foot with open eyes	Male	12	4.86 ± 1.07	-0.537	0.298	6.91 ± 3.25	-1.753	0.047
	Female	12	5.06 ± 0.72			10.98 ± 7.35		
Standing on one foot with closed eyes	Male	12	4.19 ± 0.96	-0.996	0.165	5.73 ± 1.94	-1.786	0.044
	Female	12	4.58 ± 0.93			8.43 ± 4.86		
Balance beam walking	Male	12	12.48 ± 1.03	1.042	0.154	9.00 ± 4.21	-1.736	0.048
	Female	12	12.04 ± 1.04			11.17 ± 0.98		

The *t*-value is the statistical measure of the independent sample *t*-test, and the *p*-value is the corresponding level of significance.

## 4 Discussion

This study systematically evaluated and analyzed the balance ability of 3–6 year old children through a 12 week gymnastics exercise intervention. The research results show that long-term scientific gymnastics exercises can effectively improve children’s balance ability, which has profound significance for early childhood education, physical training, and healthy growth.

### 4.1 The improvement effect of gymnastics movements on balance ability

Balance ability, as an important component of human movement ability, plays a crucial role in the stability, coordination, and overall performance of individuals (Zhang et al., 2023). Especially in the early childhood stage, the development of balance ability not only affects the mastery of motor skills, but also directly relates to the formation of daily living abilities. Therefore, how to improve children’s balance ability through effective training methods has become a focus of attention for many educators and researchers. Gymnastics movements, due to their rich content, diverse forms, and emphasis on body control and stability, have become one of the effective ways to improve children’s balance ability.

This study systematically trained the balance ability of young children by implementing a series of gymnastics interventions, including standing with closed eyes and walking on a balance beam, practicing in different positions, lifting with one foot, rolling over, trampoline, etc. The experimental results showed that after intervention in gymnastics movements, the static balance (such as standing on one foot with eyes open, standing on one foot with eyes closed) and dynamic balance (such as walking on a balance beam) abilities of the experimental group of children were significantly improved. This result is consistent with previous research and further confirms the positive impact of gymnastics movements on children’s balance ability. Specifically, gymnastics movements comprehensively exercise children’s physical coordination, flexibility, and strength through different forms of practice. In static balance exercises, such as standing on one foot with closed eyes, young children need to rely on the synergistic effect of proprioceptive and vestibular systems to maintain body balance without visual assistance. This exercise not only enhances children’s perception of their own movement state, but also promotes the brain’s processing and integration of balance information, thereby improving their static balance ability. In dynamic balance exercises such as walking on a balance beam and jumping on a trampoline, young children need to quickly adjust their body posture and position in a constantly changing external environment to maintain body balance. This exercise not only exercises children’s reaction speed and coordination, but also enhances the strength of their legs and core muscles, laying a solid foundation for improving their dynamic balance ability.

The reason why gymnastics movements can effectively improve children’s balance ability is mainly attributed to the following four aspects. One is rich in content and diverse in form. Gymnastics exercises cover various forms of practice, including standing, walking, jumping, rolling, etc., which can comprehensively exercise children’s body coordination, flexibility, and strength. Diversified exercise methods not only stimulate children’s interest and participation, but also enable them to exercise their balance ability in various sports situations. This diverse stimulation helps promote the development and connectivity of different regions of the brain, thereby enhancing the flexibility and adaptability of balance control. The second is to emphasize the control and stability of the body. Gymnastics movements require young children to maintain control and stability of their bodies during practice, constantly adjusting the position of their head and body to maintain balance. This exercise not only exercises the muscle strength and coordination of young children, but also promotes the synergistic effect of the vestibular system, visual system, and proprioceptive system. The vestibular system is responsible for perceiving the position and movement status of the head, the visual system provides visual information about the surrounding environment, and the proprioceptive system provides position and movement information of muscles, joints, and limbs. Gymnastics training enhances the functionality of these systems, improves children’s perception accuracy of body posture and movement status, and thus enhances their balance ability. Neuroscience research has shown that the enhancement of this synergistic effect is closely related to neural plasticity in regions such as the cerebral cortex and cerebellum (Jiang et al., 2016; Zha et al., 2018). The third is to promote the brain’s processing and integration of balanced information. Gymnastics movements require young children to constantly perceive and adjust their own movement state and changes in the spatial position of their heads, which requires the participation and regulation of the brain. Through gymnastics training, young children can enhance their perception of their own movement state and head position in space, promoting the brain’s processing and integration of balance information. This improvement in perception and processing abilities helps young children quickly and accurately adjust their body posture and maintain balance in complex environments. Sports physiology research has shown that gymnastics training can increase the gray matter volume and neural connections in the cerebral cortex and balance related brain regions, thereby improving the neural basis for balance control. The fourth is to enhance the psychological quality of young children. The practice process of gymnastics movements is often accompanied by certain challenges and difficulties, requiring young children to remain calm and focused in the face of difficulties and challenges, in order to overcome their inner fears and anxieties. The improvement of this psychological quality not only helps young children better cope with future learning and life challenges, but also enhances their confidence and self-esteem to a certain extent. Psychological research has shown that positive psychological states and self-confidence have a significant impact on young children’s performance in learning and life, including their ability to maintain balance.

In summary, gymnastics movements emphasize the control and stability of the body, promote the processing and integration of balance information by the brain, and enhance the psychological quality of young children through rich content and diverse forms of practice, effectively improving their balance ability. Therefore, in future early childhood education, gymnastics movements should be further promoted and applied as an effective means of balancing ability training to promote the physical and mental health development of young children.

### 4.2 Comparison with irregular physical exercise

When exploring the improvement effect of young children’s balance ability, comparing gymnastics movements with irregular physical exercise can more clearly reveal the unique advantages of gymnastics movements in enhancing young children’s balance ability. This is consistent with numerous research results both domestically and internationally. For example, numerous studies have shown that gymnastics training can significantly improve children’s balance and coordination abilities, and gymnastics movements have a positive impact on the physical and mental health of young children. However, this study is more detailed and comprehensive in the development and implementation of training plans, gradually improving children’s balance ability through phased training programs. In addition, this study emphasizes the fun and playfulness of gymnastics movements, as well as their comprehensive impact on the physical and mental health of young children, which are aspects that have been less explored in other studies.

Firstly, there are significant differences in the systematic and targeted training between gymnastics movements and irregular physical exercise. Gymnastics movements are usually based on scientific training plans and professional guidance, which can accurately train children’s balance ability. This systematic training not only ensures the comprehensiveness and depth of the training content, but also enables personalized adjustments based on individual differences of young children, thereby achieving more significant improvement in balance ability. In contrast, although irregular physical exercise can promote children’s physical development and improve their athletic abilities to a certain extent, it is often difficult to form targeted training for balance ability due to the lack of clear goals and plans. This random exercise method may lead to unstable and uneven training effects, making it difficult to achieve the comprehensive and in-depth improvement effect brought by gymnastics movements.

Secondly, gymnastics movements have the advantages of fun and playfulness. Gymnastics movements are not only scientific and systematic, but also have the characteristics of fun and playfulness. This characteristic enables gymnastics movements to stimulate children’s interest and participation in sports during the training process. Young children are often curious and enthusiastic about novel, interesting, and challenging forms of exercise, and gymnastics movements perfectly meet this need. Through the practice of gymnastics movements, young children can not only enjoy the fun of sports, but also continuously improve their physical fitness and athletic ability. This positivity and interest not only promote children’s engagement and focus in gymnastics movements, but also improve their physical fitness, athletic ability, as well as their confidence and sense of achievement in sports, laying a solid foundation for their healthy growth in the future.

Finally, gymnastics movements have a comprehensive impact on the physical and mental health of young children. Compared with irregular physical exercise, gymnastics exercises not only have a significant effect on improving children’s balance ability, but also have a positive impact on their physical and mental health. Through the practice of gymnastics movements, young children can exercise their body’s coordination, flexibility, and strength, while promoting the brain’s processing and integration of balance information. This kind of physical and mental coordination training method helps young children develop a healthy lifestyle, improve physical fitness and immunity, and reduce the risk of illness. In addition, gymnastics movements can also cultivate social skills such as teamwork, social skills, and self-management abilities in young children. During the practice of gymnastics movements, young children need to communicate and collaborate with their coaches and teammates, which helps them learn to respect, understand, and establish good relationships with others. At the same time, the training of gymnastics movements also requires young children to have a certain degree of self-management ability and self-discipline, which helps them form good study and living habits (Si et al., 2024; Zhang et al., 2012).

In summary, compared with irregular physical exercise, gymnastics movements have significant advantages in improving children’s balance ability. Gymnastics movements can be accurately trained for young children’s balance ability through scientific training plans and professional guidance; At the same time, its fun and playful characteristics can stimulate children’s interest in sports and participation enthusiasm; In addition, gymnastics movements also have a positive impact on the physical and mental health of young children. Therefore, in future early childhood education, gymnastics movements should be further promoted and applied as an effective means of balancing ability training to promote the physical and mental health development of young children.

### 4.3 There are gender differences in balance ability

Gender differences are a topic worthy of in-depth exploration in the development of children’s balance ability. Based on existing research results and the findings of this study, there are indeed gender differences in the balance ability of young children, which have different manifestations in static balance and dynamic balance (Kolic et al., 2020).

In terms of static balance, girls generally perform better than boys. This may be related to physiological characteristics such as flexibility, coordination, and balance in girls’ bodies. Girls usually have higher physical flexibility and coordination, which allows them to adjust their body posture and position more flexibly while maintaining static balance, thus effectively maintaining body stability. In addition, girls’ sense of balance may also be more sensitive, allowing them to demonstrate higher abilities in perceiving and adjusting body balance. However, in terms of dynamic balance, boys perform even better. This may be related to the athletic qualities of boys, such as strength, speed, and explosiveness. Boys usually have stronger muscle strength and higher movement speed, which allows them to react more quickly and adjust their body posture and position more effectively when facing dynamic balance challenges, thereby maintaining body balance. In addition, boys may also have stronger explosive power, allowing them to exhibit higher dynamic balance in situations that require quick movement or jumping. This discovery suggests that in the training process of gymnastics movements, targeted training and guidance should be provided based on the gender differences of young children. For girls, more attention can be paid to their advantages in static balance, and their balance ability can be further improved through training in flexibility, coordination, and balance. At the same time, some dynamic balance exercises can also be introduced appropriately to help them improve their ability to cope with complex sports situations. For boys, more attention can be paid to their advantages in dynamic balance, and their balance ability can be further improved by increasing training in strength, speed, and explosiveness. At the same time, some static balance exercises can also be introduced appropriately to help them improve their perception and control of body position and posture.

When implementing targeted training strategies, three points should also be noted. One is personalized training. Develop personalized training plans based on individual differences and gender characteristics of each child. This includes determining appropriate training intensity, frequency, and duration to ensure that every child can train in a safe and effective environment. The second is professional guidance. Provide professional guidance on gymnastics exercises to ensure that young children practice in the correct posture and technique, which helps to avoid sports injuries and improve training effectiveness. The third is continuous monitoring. Regularly monitor the development of children’s balance ability and adjust the training plan based on the monitoring results. This helps to identify and correct problems in training in a timely manner, ensuring that young children can continuously and steadily improve their balance ability.

In summary, there are indeed certain gender differences in the balance ability of young children. In the training process of gymnastics movements, targeted training and guidance should be provided based on the gender differences of young children to fully utilize their strengths and potential. By implementing personalized training strategies, providing professional guidance, and continuously monitoring the development of children’s balance abilities, we can help them better develop their balance skills and lay a solid foundation for their healthy growth.

## 5 Conclusion and suggestions

### 5.1 Conclusion

This study has drawn profound conclusions through systematic experiments and analysis. Firstly, we have clearly confirmed that long-term scientific gymnastics exercises have a significant and positive effect on improving the balance ability of 3–6 year old children, which fully demonstrates the unique value of gymnastics exercises in promoting the coordinated development of children’s bodies. Secondly, compared to irregular physical exercise, gymnastics movements have shown a more significant effect on improving children’s balance ability due to their systematic and targeted characteristics, which further highlights the important role of gymnastics movements in children’s physical training. In addition, gender differences in children’s balance ability have been found, suggesting that when conducting gymnastics training, we should fully consider the gender characteristics of children and implement more targeted training strategies and guidance methods. These conclusions not only greatly enrich the research field on the impact of gymnastics movements on children’s balance ability, but also provide solid scientific basis and practical guidance for the overall planning of early childhood education, physical training, and even the healthy growth of young children.

### 5.2 Suggestions

Based on the above conclusions, this study proposes the following suggestions to promote the effective promotion and implementation of gymnastics movements in young children, so that gymnastics can better serve the healthy growth of young children.

#### 5.2.1 Suggestions from relevant government departments

The relevant government departments should play a key role in promoting and implementing gymnastics movements among young children. Specifically, the government should establish a special fund aimed at supporting the research and development, promotion, and teaching activities of gymnastics movements, in order to promote the scientific optimization of gymnastics movements, improve teaching quality, and expand the influence of gymnastics among young children. The expected goal is to make gymnastics one of the compulsory physical education contents in kindergartens and primary schools nationwide, significantly improving children’s balance ability and physical fitness. At the same time, the government should explicitly require educational institutions such as kindergartens and primary schools to officially include gymnastics movements in their physical education curriculum, ensuring that young children can receive systematic training under professional guidance, and developing corresponding policies and standards to guide schools to scientifically and reasonably arrange gymnastics curriculum content, effectively improving their balance ability, physical fitness, and sports skills (Tao and Yuan, 2024). In addition, the government should strengthen social propaganda, widely disseminate the positive impact of gymnastics movements on the physical and mental health of young children through various platforms such as media and the internet, organize gymnastics activities in schools and communities, invite gymnastics champions or experts to give lectures and demonstrations, in order to enhance parents’ and teachers’ awareness and attention to such movements.

#### 5.2.2 Suggestions from the school

As an important place for training young children’s gymnastics movements, schools should fully leverage their professional and educational advantages (Yin and Ma, 2020). Specifically, schools should design suitable gymnastics courses for children of different age groups based on their growth and development characteristics, such as designing simple balance beam walking and basic gymnastics movements for 3–4 year old children, adding jumping, rolling and other movements for 5–6 year old children, and paying attention to the accuracy and fluency of movements. At the same time, schools should innovate teaching methods, cleverly combine gymnastics with games, and design creative and interesting gymnastics games, such as balance beam relay races, gymnastics ball throwing, etc., to enhance children’s hand eye coordination ability and physical fitness. In addition, schools can integrate elements such as music and dance into gymnastics teaching, making gymnastics more lively and interesting, and stimulating children’s participation and interest. To ensure teaching quality, schools should also strengthen teacher training, regularly organize teachers to participate in professional training on gymnastics movements, improve their professional competence and teaching level, and provide strict training and management for preschool gymnastics coaches to ensure that they master scientific and systematic training methods and have professional guidance abilities.

#### 5.2.3 Suggestions for teachers

Teachers play a crucial role in teaching gymnastics movements, and should continuously enrich teaching methods by designing a series of creative and interesting gymnastics movements and combinations, such as imitating animals, cartoon characters and other vivid gymnastics movements, and combining teaching methods such as stories and scenarios to make gymnastics teaching more lively and interesting (Chen, 2019). At the same time, teachers should flexibly use incentive mechanisms to encourage young children to actively participate in gymnastics training and competition activities through rewards, praise, and other means, such as setting up awards such as “Gymnastics Little Star” and “Best Progress Award” to stimulate children’s enthusiasm and confidence in participation. In addition, teachers need to pay attention to the individual differences of young children and tailor personalized training and guidance plans based on each child’s physical condition, balance ability, and interest characteristics. For children with relatively weak balance ability, appropriate strategies such as reducing training difficulty and increasing auxiliary exercises should be adopted to gradually help them improve their balance ability.

#### 5.2.4 Suggestions for parents

Parents play an indispensable role in the learning and training of gymnastics movements for young children. In order to support their children’s gymnastics learning, parents should prepare simple gymnastics exercise equipment and suitable venues at home, such as balance beams, gymnastics mats, etc., to create a safe and comfortable practice environment, and encourage children to develop the habit of independent practice. In addition, parents should actively encourage their children to participate in formal gymnastics training classes or clubs, maintain close communication with coaches, and jointly pay attention to their children’s training progress and areas for improvement. More importantly, parents should actively participate in their children’s gymnastics activities, watch gymnastics competitions and participate in gymnastics training with their children, enhance family harmony through parent–child interaction, and set an example by practicing gymnastics movements with their children, inspiring their love and perseverance in gymnastics, and enjoying the fun and sense of achievement brought by gymnastics together (Yu et al., 2022).

## Data Availability

The original contributions presented in the study are included in the article/supplementary material, further inquiries can be directed to the corresponding authors.
